# Compressed sensing cardiac MRI exploiting spatio-temporal sparsity

**DOI:** 10.1186/1532-429X-15-S1-E14

**Published:** 2013-01-30

**Authors:** Jafar Zamani, Abbas N Moghaddam, Hamidreza Saligheh Rad

**Affiliations:** 1 Biomedical Engineering, Amirkabir University of Technology (Tehran Polytechnic ), Tehran, Islamic Republic of Iran; 2 David Geffen School of Medicine, UCLA, Los Angeles, CA, USA; 3 Medical Physics and Biomedical Engineering, Tehran University of Medical Sciences, Tehran, Islamic Republic of Iran; 4 Quantitative MR Imaging and Spectroscopy Group, Research Center for Molecular and Cellular Imaging, Tehran University of Medical Sciences, Tehran, Islamic Republic of Iran

## Background

Compressed Sensing (CS) is a theory with potential to reconstruct sparse images from a small number of random acquisitions. Particularly in MRI, CS aims to reconstruct the image from incomplete K-space data with minimum penalty on the image quality. The image is recovered from the sub-sampled K-space data, using image sparsity in a known sparse transform domain. Cardiac MRI has a sparse structure in both temporal and spatial domains; making CS a promising method for such application.

## Methods

Experiments were performed on a data set acquired by Cagdas Bilen *et al.*[1]. Fully sampled data were acquired using a 128×128 matrix (FOV = 320 × 320 mm) and 23 temporal frames covering the cardiac cycle. In this study, we reconstructed eight (one in every three) frames through CS using Gradient Projection for Sparse Reconstruction (GPSR) algorithm. The remaining 15 frames were reconstructed through a combination of CS and temporal information (TI). Sampling rate for the CS and CS-TI frames was set to 0.5 and 0.3, respectively. Block Discrete Cosine Transform (BDCT), Block Walsh-Hadamard Transform (BWHT) and Gaussian Transform were used to create measurement matrix in CS. Discrete Wavelet Transform (DWT) was used as sparse basis. The fidelity term in cost function is modified as: g=0.9||Fu m-y||2+0.1||TE-m||2, where Fu represents the under-sampled Fourier operator, y represents the K-space under-sampled data, and TE (Temporal Estimation) represents the obtained frames from TI. In this study, we use interpolation (I), forward motion estimation (FME) and forward-backward motion estimation (F-B ME), respectively on previous and next CS frames to obtain TI.

## Results

**Figure 1:**
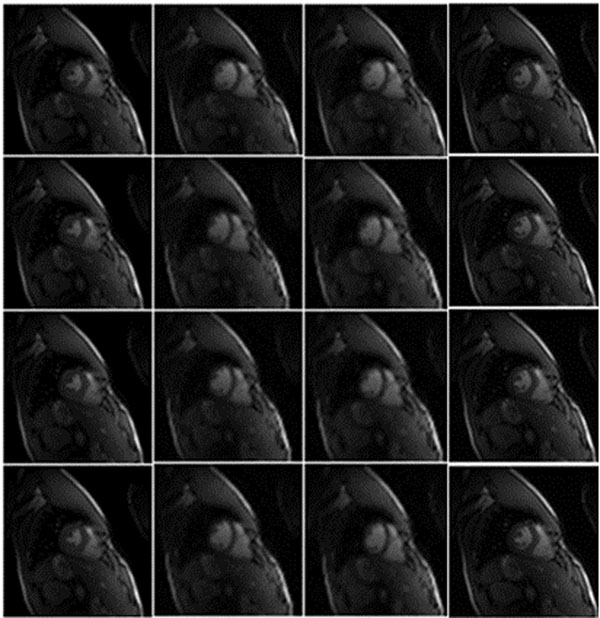
From left to right, First row) original image, CS frame with BWHT, BDCT and Gaussian measurement matrices. Second row) CS-TI using I method frame with BWHT, BDCT and Gaussian measurement matrices. Third row) CS-TI using FME method frame with BWHT, BDCT and Gaussian measurement matrices. Fourth row) CS-TI using (F-B) ME method frame with BWHT, BDCT and Gaussian measurement matrices.

Figure 1 illustrates one frame from the original set along with the corresponding CS and CS-TI frames reconstructed with proposed methods for TI generation. Table 1 shows numerical results including SNR, PSNR, Structural SIMilarity (SSIM) and computational time for each proposed method.

**Table 1:** Results Of Proposed Methods Show That BWHT Outperform Other Methods.

Measurement Matrix	methods	TI - Methods	SNR	PSNR	SSIM	Time(s)
	CS		26.750626	82.80	0.981008	11.3048
	
		I	28.076336	83.82	0.983096	2.5165
		
BWHT	CS-TI	FME	27.796233	83.88	0.974924	2.1939
		
		F-B ME	27.946202	83.93	0.979615	2.3913

	CS		21.545575	75.25	0.827131	12.1182
	
		I	22.505806	76.34	0.780809	2.9337
		
Gaussian	CS-TI	FME	20.527749	72.95	0.791594	2.9910
		
		F-B ME	21.586516	76.32	0.805057	3.0172

	CS		26.985696	82.07	0.974128	12.6598
	
		I	27.656441	83.16	0.958932	2.8911
		
BDCT	CS-TI	FME	27.720514	83. 87	0.977975	2.3867
		
		F-B ME	29.923755	83.86	0.962291	2.1828

## Conclusions

The proposed method increased under-sampling rate and expedited reconstruction time in CS theory. The results were quantified using SNR, PSNR and SSIM for the quality of the reconstruction and the computational time, concluding that BWHT outperforms other methods in both quality measures and computational time with 15% and 10%, respectively. In all aforementioned a derivative of the proposed method, the processing time was at least 4 times accelerated compared to the routine CS algorithm.
